# Dynamic of the structural alteration of biochar in ancient Anthrosol over a long timescale by Raman spectroscopy

**DOI:** 10.1371/journal.pone.0229447

**Published:** 2020-03-23

**Authors:** Daniel Vieira de Sousa, Luciano Moura Guimarães, Jorlandio Francisco Félix, João Carlos Ker, Carlos Ernesto R. G. Schaefer, Maria Jacqueline Rodet

**Affiliations:** 1 College of Geography, Federal University of São Francisco Valley, Senhor do Bonfim, Bahia, Brazil; 2 Physics Department, Federal University of Viçosa, Viçosa, Minas Gerais, Brazil; 3 Institute of Physics, University of Brasilia, Brasilia, Federal District, Brazil; 4 Department of Soils, Federal University of Viçosa, Viçosa, Minas Gerais, Brazil; 5 Archaeology Department, Federal University of Minas Gerais, Belo Horizonte, Minas Gerais, Brazil; RMIT University, AUSTRALIA

## Abstract

The presence of biochar with high carbon accumulation capacity and nutrient adsorption is causally associated with archeological soils. Although this type of soil organic matter has been known for a long time, the knowledge of its structure and environmental behavior is still limited. This work used Raman spectroscopy to obtain structural information and identify alterations in biochar particles. To this end, we studied biochar particles found in an archaeological site with a temporal window lasting 12451 to 11080 yr cal BP. The molecular, structural and sp2/sp3 characteristics of the charcoal particles were determined at the time of burning and associated with the temperature, time and characteristics of the burnt material. We propose that the process of oxidation of the biochar occurs during the first 2000 years after its genesis. The oxidation process is a reflection of decreases in the number of defects related to sp2 bonds on amorphous carbons and increases in the number of defects associated with ionic impurities, which clearly indicate the interaction between biochar particles and the soil matrix. The data confirm the hypothesis that the persistence of biochar in the environment is due to its graphite structure and suggest that over a 12000 year timeframe, biochar particles undergo several changes that occur in the disordered phase and are rapidly oxidized.

## Introduction

Biochar can be conceptualized by two distinct perspectives: the “chemical” perspective and the "utility" perspective. In the “chemical” perspective, biochar can be understood as the solid product of biomass pyrolysis that formed by the incomplete combustion of organic compounds [1–3] and is composed of a graphite microstructure with ordered and disordered phases [3]. The ordered phase is attributed to polyaromatic nanographite domains, which confer structural stability and long-term permanence in the environment. The disordered/amorphous phase is the most susceptible to changes. This phase is composed of carboxylic and aliphatic groups [4, 5], which are responsible for generating electrical charges and adsorbing nutrients [6, 7]. According to the modern concept, which relates to "utility" and intentionality, biochar is widely recognized as the appropriate term for man-made charcoals that are used to improve the chemical and physical properties of soil [8].

One early focus of biochar studies was on the anthropic soil of the Amazon forest known as "Indian Black Earth," which is found in open-air archaeological sites with human occupation dating between 800 and 2000 years before the present (BP) [9–12]. Despite the numerous studies on biochar, its stability and dynamics in the environment have yet to be fully elucidated, and this question has been examined and discussed for decades [13–17]. Previous researchers clarified that a higher burning temperature produces biochar with higher polyaromatic structures and larger crystallite size.

Recent studies have shown that biochar particles are the altered (oxidized) in soil and sediments, revealing that biochar can be altered over time through biotic and abiotic mechanisms [18–20]. To further elucidate the process and mechanisms of biochar degradation, it is necessary to evaluate the structural alterations in detail over time, and this line of inquiry is particularly important in the context of carbon sequestration and global warming mitigation as well as for biochar transport, erosion, cation retention, and stability [21–24]. Previous studies have addressed the structural alteration of biochar particles using a time scale analysis, although the time scales of these studies did not exceed 1000 years [18, 20, 25–27]. An approach that covers a longer timescale (Pleistocene/Holocene to historical period) in the same locality is still unprecedented in biochar research.

A considerable variety of methods have been used to reveal the structure of biochar, including NMR, FTIR, XPS, SEM-EDS, Synchrotron-based techniques and Raman spectroscopy [8, 28–31]. Raman spectroscopy is very sensitive to alterations in the structure of carbon and therefore is able to shed light on the degradation of biochar in soil. This technique has been used for studies of the nanostructures of charcoal (biochar) in Anthrosols [4, 20, 32, 33].

The most prominent features in the Raman spectra of graphite materials are the so-called G and D bands. The G band is the signature of graphite materials associated with the vibration of the carbon atoms tangentially to the plane of the graphene. This band can be observed at approximately 1580 cm^-1^, and it indicates the presence of organized sp2 domains [34–37]. The D band is related to defects associated with the breaking of the hexagonal symmetry of the carbon atoms in the graphene sheets and can be observed at approximately 1350 cm^-1^. The relationship between the intensities of the D and G bands can provide an estimate of the crystallite size and also related to the number of defects present in the material [36]. However, for highly disordered materials, other bands are induced by other types of defects in the crystalline lattice and appear as first-order peaks at approximately 1150 (D4), 1530 (D3) and 1600 cm^-1^ (D2) [25].

To elucidate the biochar degradation across the time, we examine the biochar particles over a long timescale in an ancient anthropic soil using Raman spectroscopy to evaluate the alteration of the structure of biochar over time. Our study adopted an original and previously unpublished approach that considers a long temporal period (e.g., the Pleistocene/Holocene 12702 yr cal BP to the historical period). This approach helps to understand the behavior of biochar over time and provides information on the dynamics of changes of the carbon particles in the environment.

This research is based on the ancient anthropic soil found at the Bibocas II archaeological site (UTM coordinates 563175, 8096575) located in the municipality of Jequitaí, and it is approximately 34 m long and 5 m deep. This site is located in the central Brazilian Plateau in the upper São Francisco River basin between the archaeological provinces of Lagoa Santa and Peruaçu Valley, which are the regions in Brazil with the most archaeological sites dating from the late Pleistocene to the early Holocene [38, 39]. According to Sousa [40, 41], anthropic action in the Bibocas II archaeological site is responsible for the high concentrations of carbon, calcium, phosphorous, and potassium and the high magnetic susceptibility. The anthropic activities in the site influenced the melanization of the soil, mainly in the stratigraphic layers, where bonfires have been identified. Melanization is a product of higher organic matter and charcoal contents. Sousa [40] observed a close correlation between the amounts of carbon, potassium, and calcium and magnetic susceptibility and suggested that magnetic susceptibility could result from intense heating of the soil caused by anthropogenic fires. Such in situ human activity would promote the formation of new mineral phases, such as magnetite and maghemite [41].

Current regional research is concentrated on the banks of the São Francisco River and in the karst regions of Jequitaí and Lagoa dos Patos [42, 43], and the results show that the local prehistoric people produced very elaborate unifacial and bifacial lithic instruments from the various raw materials available in the local environment (e.g., quartzite, quartz, silexite, and green chloritic rocks). The essential element of these cultures was the systematic pebble debitage, which produced rock flakes that were directly used without further transformation [42]. The evidence of this technological choice is present throughout the stratigraphic sequence up to the time of contact with the European colonizers, which occurred at the São Francisco riverbank sites. Fires used to cook food or transform raw materials for painting are a constant regional feature. Some shelters in the Peruaçu River valley of the Itacarambi municipality have remnant bonfires that were used only for making pigments. In the Bibocas II archaeological site, bonfires are systematically identified throughout the excavated stratigraphy (in all the layers) and represent the origin of the charcoals used in this research.

This shelter was chosen for this study for some reasons: i) it has thick, continuous and undisturbed sedimentary sequences and ii) it shows evidence of old occupation, with ^14^C dating (12451 to 11080 yr cal BP [Beta-265452]) indicating continuous human occupation up to the present day [42].

Our primary objective was to investigate the changes in molecular structures of charcoal (biochar) over a long timescale of natural oxidation. The questions that drive the present study are as follows: Assuming that charcoal particles, although stable, change over time, how do these changes happen over a long time scale? What contribution can Raman spectroscopy bring to the process of charcoal particle alteration?

## Materials and methods

### Charcoal production in muffle ovens

A higher pyrolysis temperature of carbon-related material generates a more organized carbon structure, which is a reflection of the greater amount of the graphite component compared to that in the disorganized phase [3]. To generate charcoals with a known structural organization that can be used as standard material, charcoals were produced using muffle ovens at different pyrolysis temperatures.

It is important to highlight that the landscape of the archaeological site location underwent modifications at the end of the Pleistocene and during the Holocene, and these modifications led to alteration of the structural vegetation. Thus, with the exception of the more recent archaeological layers, it is impossible to use plant materials to produce these charcoals, which resemble the materials used by prehistoric populations. Because these charcoals are only included to provide a gradient of samples with distinct structural organizations, which is strongly influenced by the pyrolysis temperature [3, 8, 36, 44], any plant material could be used.

Therefore, eucalyptus bark was chosen because it is a very available residue from Brazil and has great potential for use in the production of biochar. Thus, by characterizing the structural organization of biochar produced from eucalyptus bark, we contribute relevant information about the temperature and time of pyrolysis and the respective structural organization of the material. The eucalyptus peels were dried in a forced-air circulation oven at 70 °C until reaching a constant mass and then passed through a 2 mm mesh to obtain a homogeneous sample. Pyrolysis was conducted in a muffle furnace under atmospheric air with samples and then conditioned in closed porcelain crucibles. Pyrolysis temperatures of 300, 400, 550, 700, and 1000 °C were applied for 10 minutes. The heating rate was 10 °C/minute until reaching the final temperature.

### Archaeological samples: Collection and sample preparation

The archaeological field work carried out at the Bibocas II was coordinated by the archaeologist Maria Jacqueline Rodet, who obtained permission from the Institute of National Historical and Artistic Heritage (IPHAN) to carry out the intervention in this archaeological site as well as research that led to the excavation work. During the field season, soil samples were collected at all stratigraphic layers.

**Fig 1:**
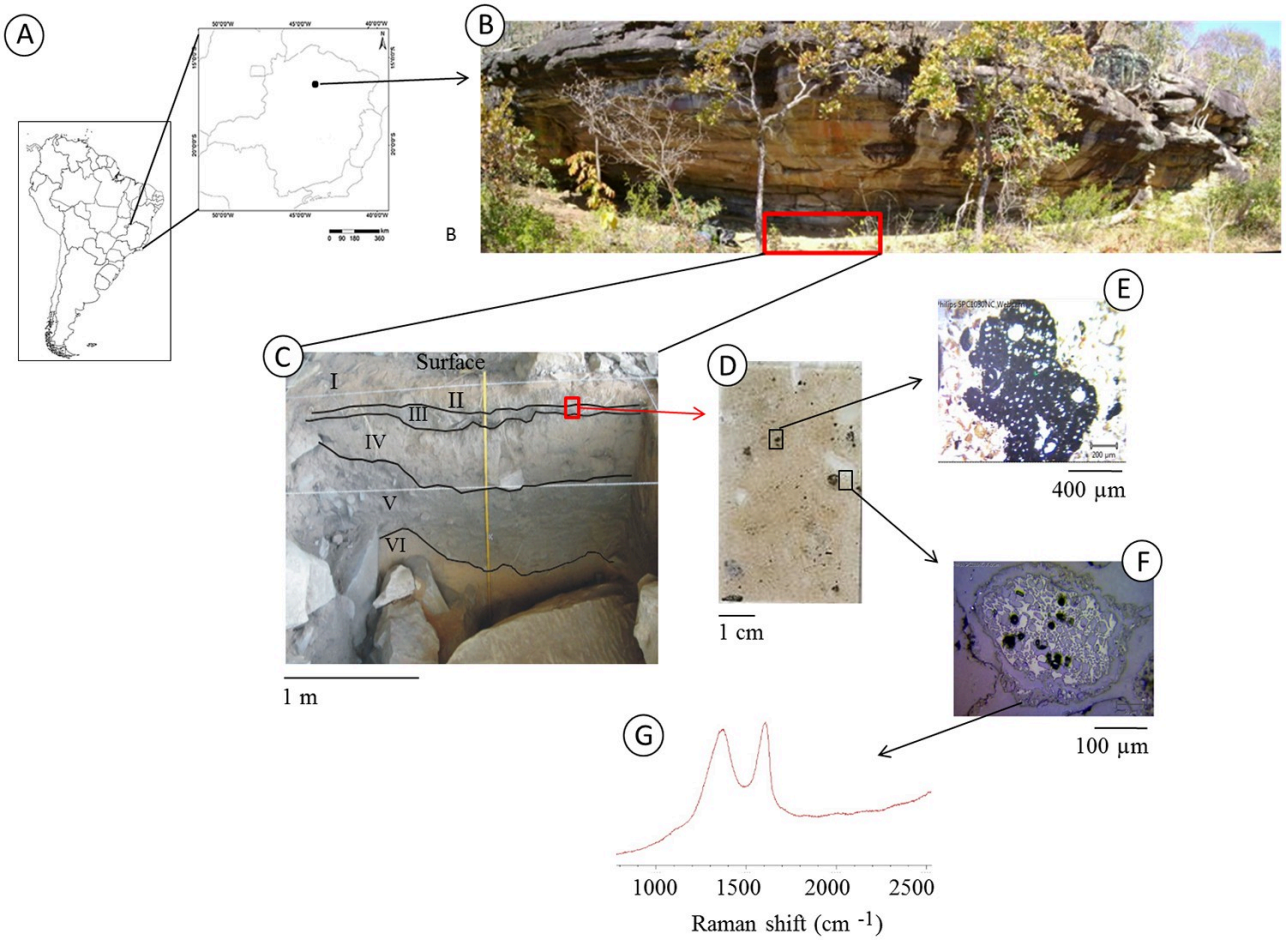
From the meter (landscape and archaeological site) to the micrometer (charcoal particle) scale. A—Location of the sample collection area in South America. B—Archaeological site Bibocas II. The area marked with the red box indicates the excavated site. C—Stratigraphy of the archaeological site with the dating. The black box indicates the location of the thin section samples. D—Micromorphology thin section. The black boxes indicate the locations in which the carbon particles in the transect were analyzed. E—Larger particle of charcoal observed with an optical microscope with transmitted light. F—Smaller particle of charcoal observed with an optical microscope with incident light. G—Example of a Raman spectrum, still untreated, of the smaller-sized charcoal particle (image F).

To compose the “charcoal sample” analyzed in this work, three charcoal particles (biochar) were randomly collected from the coarse sand fractions (in 1.5 mm to 4 mm) of the following stratigraphic layers: Surface, with human occupation from the early 20th century; Layer I, III (1698 to 1656 yr cal BP [Beta-256643]), and Upper IV; Middle IV, Lower IV, and Middle V (8543 to 8357 yr cal BP [Beta-256648] and 10,230 to 9885 yr cal BP [Beta-256648]); Lower V (11090 to 10,655 yr cal BP [Beta-264540]); and Upper VI (12702 to 12543 yr cal BP [Beta-256647]) (Fig 1 and Table 1). The uncalibrated ^14^C dates were measured at the Beta Analytic Laboratory [42]. The calibrated ages were obtained using OxCal [45]; https://c14.arch.ox.ac.uk/oxcal/OxCal.html) and the SHCal13 Southern Hemisphere calibration dataset [46].

**Table 1:** Samples used in this work.

Sample	Heat Treatment (°C)	Age ^14^C			Sample (ID)
		Calibrated Age	Uncalibrated Age	Laboratory Code	
Eucalyptus bark	400		Actual		400
Eucalyptus bark	550		Actual		550
Eucalyptus bark	700		Actual		700
Eucalyptus bark	1000		Actual		1000
Surface	--		Actual		0
Layer I	--		Nd *		1
Layer III	--	1698 to 1656	1670+50	Beta-256643	2
Layer IV upper	--		Nd *		3
Layer IV middle	--		Nd *		4
Layer IV lower	--		Nd *		5
Layer V middle	--	8543 to 8357; 10230 to 9885	7610±50; 8950±50	Beta-56648; Beta-256646	6
Layer V lower	--	11090 to 10655	9560+50	Beta-264540	7
Layer VI	--	12451 to 11080; 12702 to 12543	10440±80; 10670±50	Beta-265452 Beta-256647	8
Fragment of charcoal in Layer III micromorphology thin section of Layer III (size of charcoal 1.5 mm)	--		Nd *		
Fragment of charcoal in Layer III micromorphology thin section of Layer III (size of charcoal 600 μm)	--		Nd *		

* not dated

The collected charcoals were cleaned with deionized water and dried at 40 °C. Glass microscope slides were used to deposit these samples for Raman spectroscopy measurements. During the fieldwork, undisturbed samples (micromorphology samples) were collected between Layers II and III to prepare soil thin sections. These undisturbed samples were impregnated with an epoxy resin [47]. Two charcoal fragments on soil thin sections were selected for the point-to-point analysis, which was performed from the edge toward the center of the charcoal particles.

### X-ray diffraction

An X-ray diffraction analysis was used to study the crystallinity of samples submitted to different thermal treatments. The diffraction patterns were obtained using a Bruker D8 Discover diffractometer equipped with Cu Kα radiation (λ = 1.5418 Å). The diffraction pattern was obtained at diffraction angles between 5 and 70° (2θ) at room temperature with a scan velocity of 0.02°/s.

### Raman spectroscopy and spectrum adjustment procedures

Raman measurements were carried out on a Renishaw micro Raman inVia spectrometer. An argon laser at 514.5 nm was used as an excitation source. The spectra were obtained using a 50X objective and NA = 0.75 with a laser power of 0.3 mW on the sample, which resulted in a spot diameter of approximately 1 μm. For each sub-sample, 5 Raman spectroscopy measurements were performed, totaling 15 measurements per sample.

**Fig 2:**
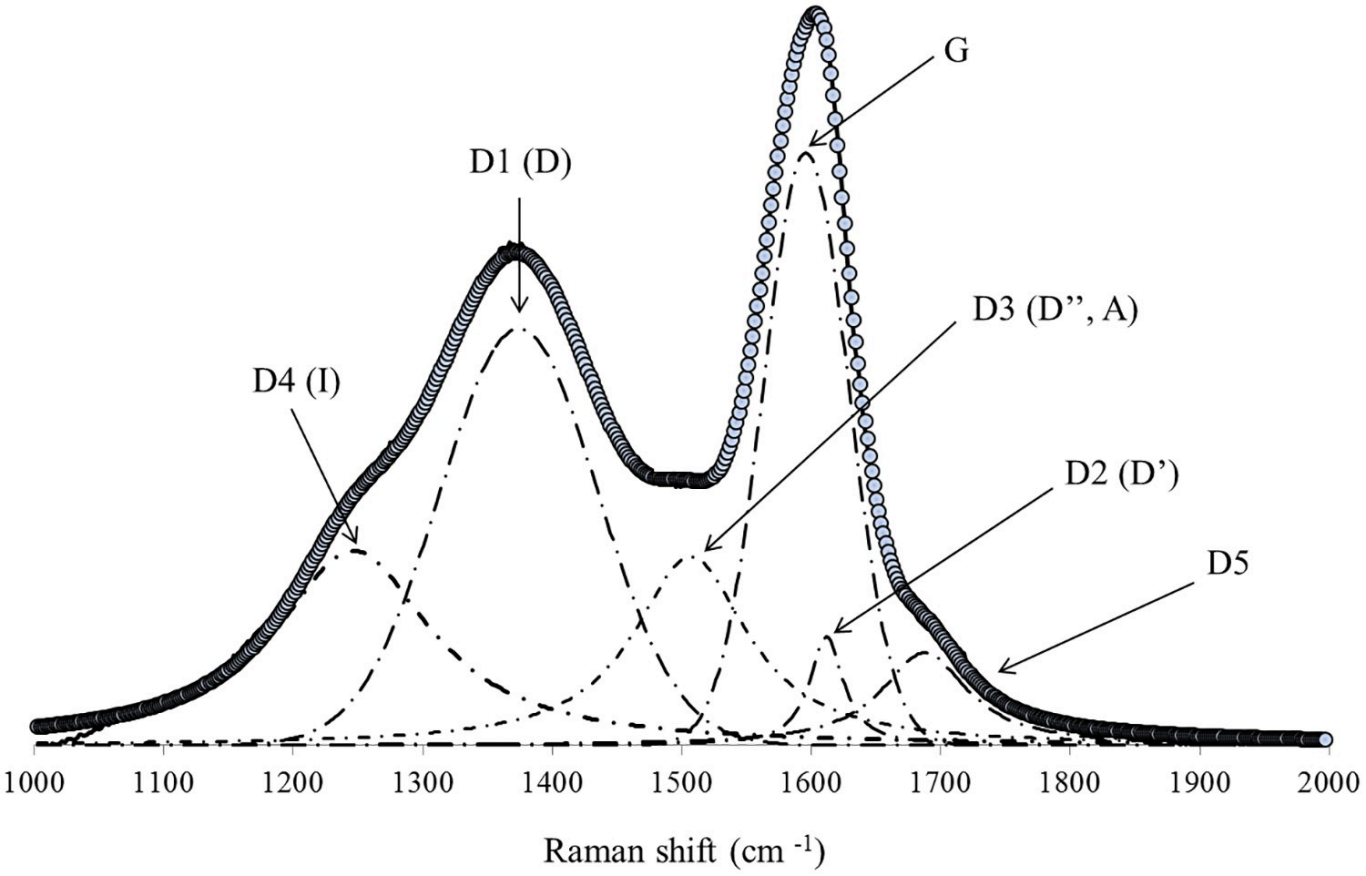
Example of the fitting performed in the samples used in this work.

The baseline of the spectra was extracted via a linear function. The spectrum fit was performed with 6 curves: two Gaussian (D and G) and four Lorentzian functions (D2, D3, D4, and D5). In general, the deconvolution of the spectrum can be performed by simple adjustment using only two Lorentzian or two Gaussian functions, which allows for the study of the dispersion behavior of the D and G Raman bands without a high level of details [48]. However, when the primary aim of the research is to identify the subtle nuances of the spectrum, a 5-band (G, D1, D3, D3, and D4, see Fig 2) adjustment is required [12, 25, 49–55]. The D5 band at 1700 cm^-1^ was inserted to improve the accuracy of the fit. Table 2 shows general information about each of the bands (Fig 2).

**Table 2:** General information on the bands used in this work.

Band	Raman Shift (cm-1)			Vibration Mode	Phonon Type	Adjust Model	Source
	Initial Position in Carbon Material						
	Soot	Disordered Graphite	HOPG				
G	1580, s	1580, s	1580, s	Ideal Graphite Lattice (E2g –Symmetry) sp2 in rings and chains	iTO, LO	Gaussian	Tuinstra F, Koenig (1970) [34]; Wang Y. et al. (1990) [37]; Ferrari & Robertson (2000) [48]; Dresselhaus et al. (2005) [35]
D1 (D)	1350, vs	1350, m	-	Discorded graphite lattice (A1g-Symmetry) sp2 in rings	iTO	Gaussian	Tuinstra F, Koenig (1970) [34]; Wang Y. et al. (1990) [37] Dresselhaus et al. (2005) [35]
D2 (D’)	1620, s	1620, w	-	E2g Simetry	LO	Lorentzian	Wang Y. et al. (1990) [37]
D3 (D”, A)	1500, m	-	-	Amorphous Carbon		Lorentzian	Jawhari T. et al. (1995) [49]; Wang Y. et al. (1990) [37] Dippel B. et al. (1999) [51]
D4 (I)	1180, w	-	-	Disordered carbon (A1 symmetry) polyenes, ionic impurities sp2-sp3, or C = C or C-C stretching		Lorentzian	Al-Jishi R & Dresselhaus G (1982) [50]; Cuesta et. al. (1998) [52]
D5	1700, w			C = O		Lorentzian	Inoue et al. (2017) [20]

s–strong; vs–very strong; m–medium; w—weak;

The crystallite size (La) calculation was performed using the Tuinstra and Koening equation, in which La = A x (I_G_/I_D_), where “A” is a constant that depends on the energy of the laser. For the 514.5 nm laser line, “A” is equilibrated at 44 Å [34].

### Scanning electron microscopy

Samples of soil thin sections were coated with graphite and analyzed by scanning electron microscopy using a JEOL model 6010LA coupled to a dispersive energy spectrometer (EDS). The measurements were performed at 15 kV.

### Statistical analysis

Multivariate statistical analyses (cluster analysis (CA) and principal component analysis (PCA)) were performed to find patterns in the structural alteration data. CA employed the amalgamation rule of “single linkage” with the “Euclidean distance.” The PCA was based on the correlation matrix. Statistica 7.0 software was employed for the statistical analyses. The CA and PCA were performed using the following variables: ωG; ωD; the ratio of the area of the D1 band and G band (AD1/AG) and the ratio of the intensity of the D1 and D4 band.

## Results

### Structural characteristics of standard charcoal pyrolyzed in muffle ovens

**Fig 3:**
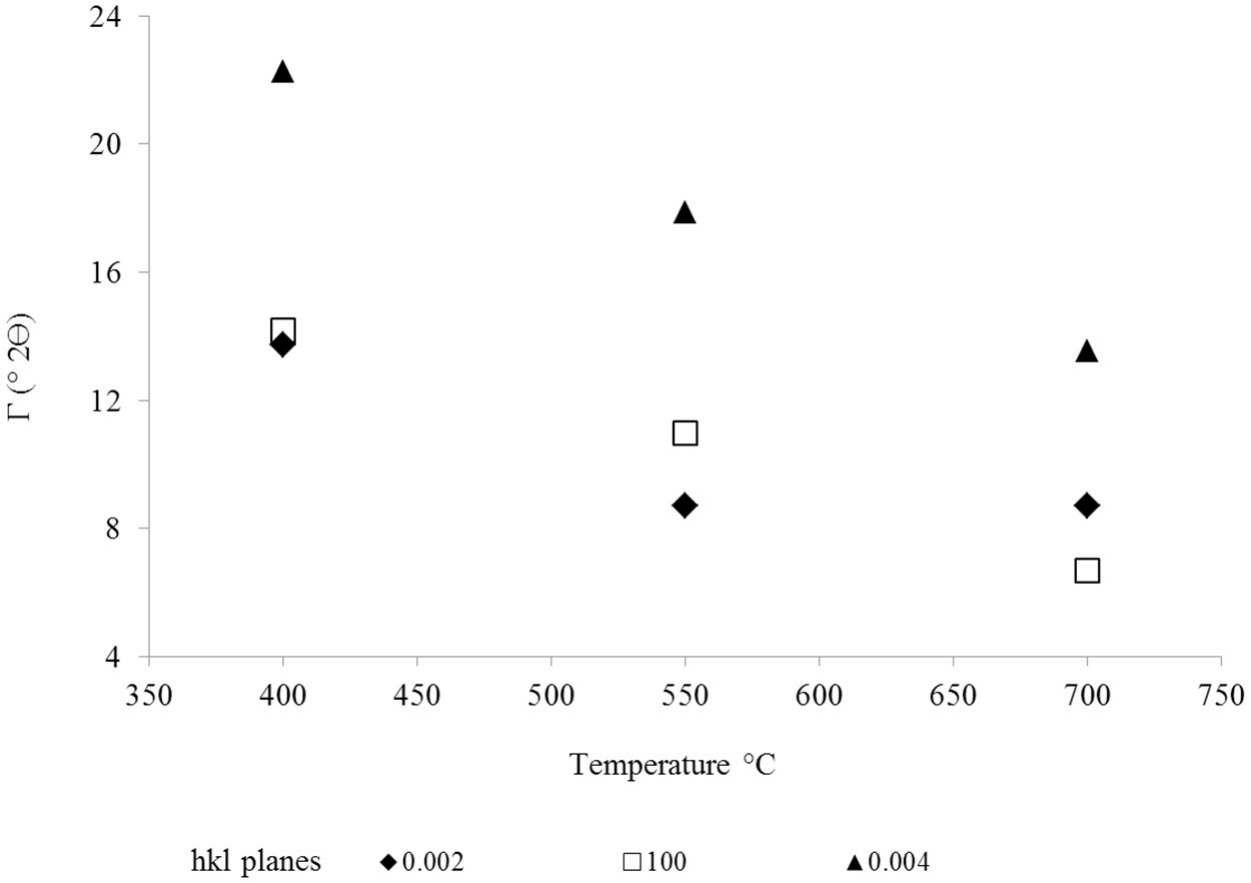
Width of the base of the peak to half-height in 2Ɵ for the hkl planes (002), (004), and (100).

The X-ray diffraction (XRD) analyses (Fig 3) revealed the changes of the biomass during the pyrolysis reaction and showed that the full width half maximum (FWHM) for the lattice planes (hkl planes (002), (004), (100)) decreased with increasing temperature.

**Fig 4:**
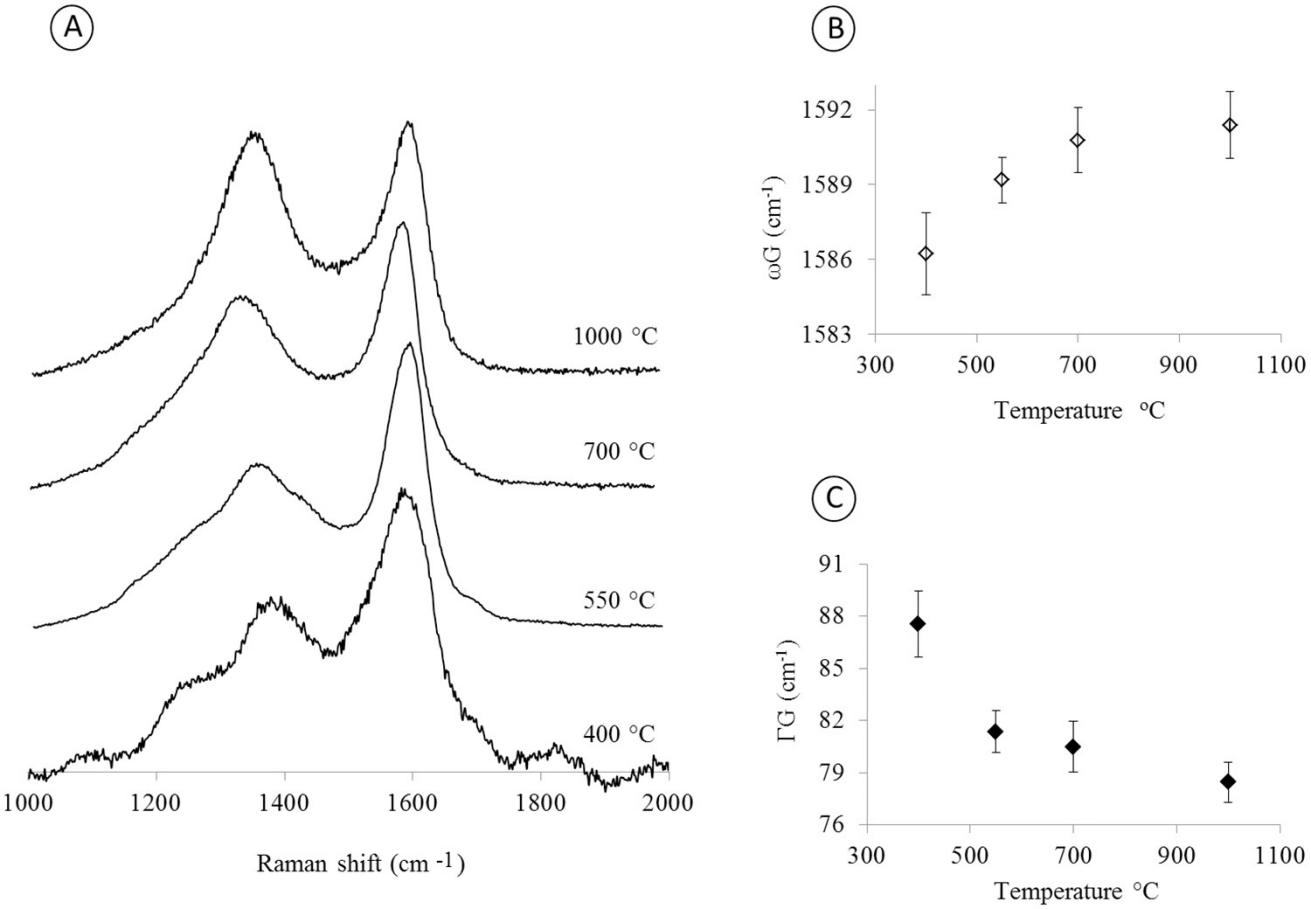
(A) Raman spectra of the charcoal samples obtained from the eucalyptus bark burned at temperatures from 400 to 1000 °C; (B) FWHM for the G-band as a function of the firing temperature; and (C) frequency of the G-band as a function of the charred temperature.

Fig 4A shows the Raman spectra for the charcoal produced samples at several temperatures and illustrates the reorganization of the lignocellulosic structures in polyaromatic structures with the subsequent organization of graphite structures. This process can be observed by the appearance of the G band in the Raman spectrum. This reorganization is caused by the charring of the vegetal biomass [55, 56].

The G-band is a signature of the sp2-type carbon structure. The D band arises due to the broken symmetry of the hexagonal structure of the carbon atoms caused by defects in the lattice, which may be a simple vacancy to the replacement of atoms in the sp2 hybrid orbitals. A G-band shift from 1584 cm^-1^ to 1592 cm^-1^ was observed prominently between 400 and 700 °C (Fig 4B). The FWHM peak of the G-band decreases as a function of the temperature (Fig 4C), which indicates an enhancement of the crystallinity and is consistent with the XRD results shown in Fig 3.

### Structural differences between pyrolyzed fresh charcoals and “fossil” charcoal

**Fig 5:**
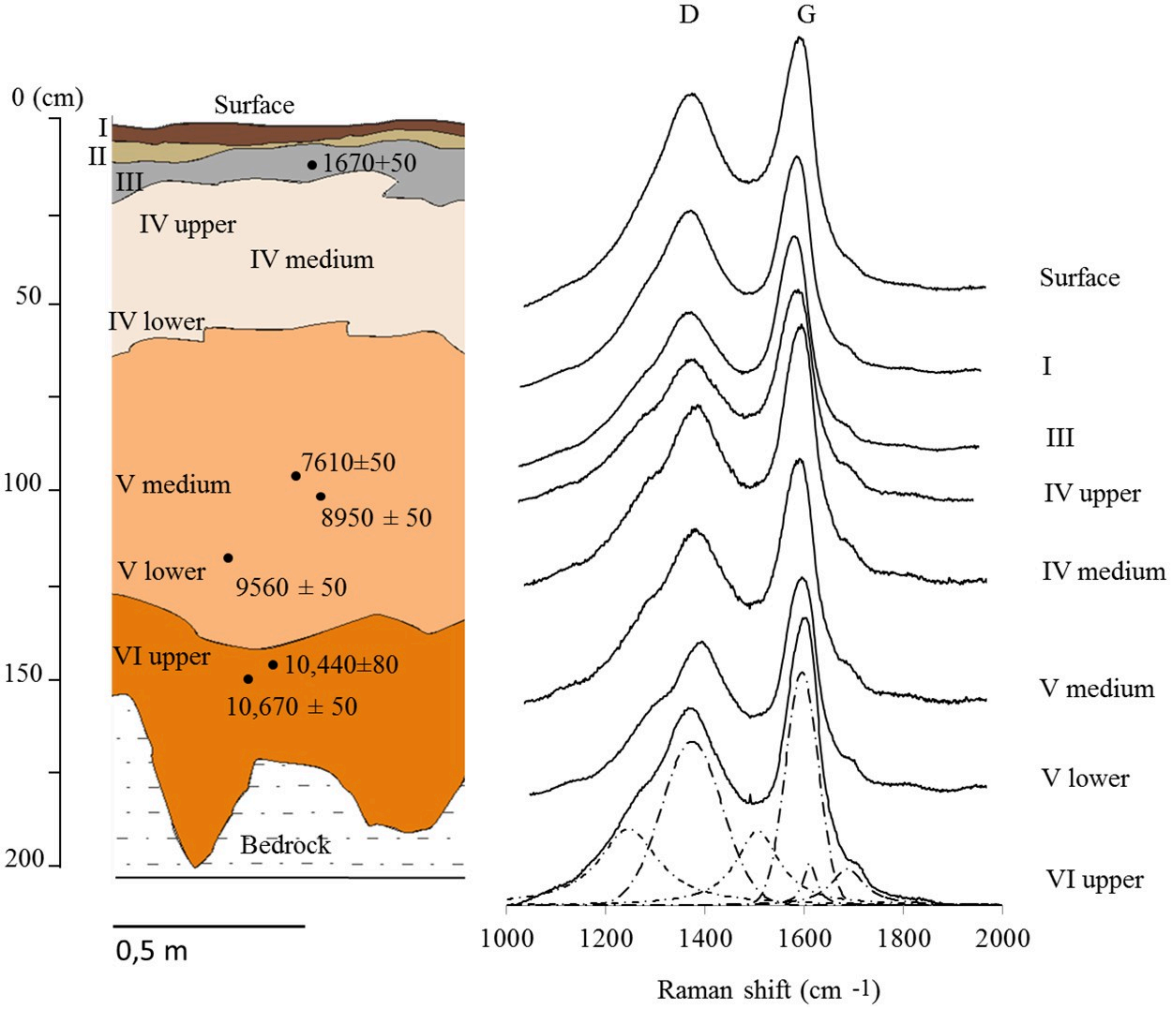
Schematic profile of the stratigraphy of the archaeological site Bibocas II by Déborah Duarte and Luis Felipe Bassi (A). Raman spectra of charcoal superimposed according to stratigraphy (B).

The stratigraphic profile of the Bibocas II archaeological site is represented by the respective layers shown in Fig 5A. The Raman spectra of the samples of charcoal collected in these different layers are visualized in Fig 5B. Fig 5B shows that there is a subtle increase of the D4 band as it deepens in the soil profile. The origin of this band may be related to the increase of structures with sp2-sp3, C-C or C = C bonds; this band is associated with defects of the sp2 network. The D1 band becomes less intense, and the peak relative to the D4 band (Fig 5B) becomes stronger. This behavior makes the D4 band more evident in the spectrum, which can be explained by the interaction between the biochar particles and the soil matrix.

**Fig 6:**
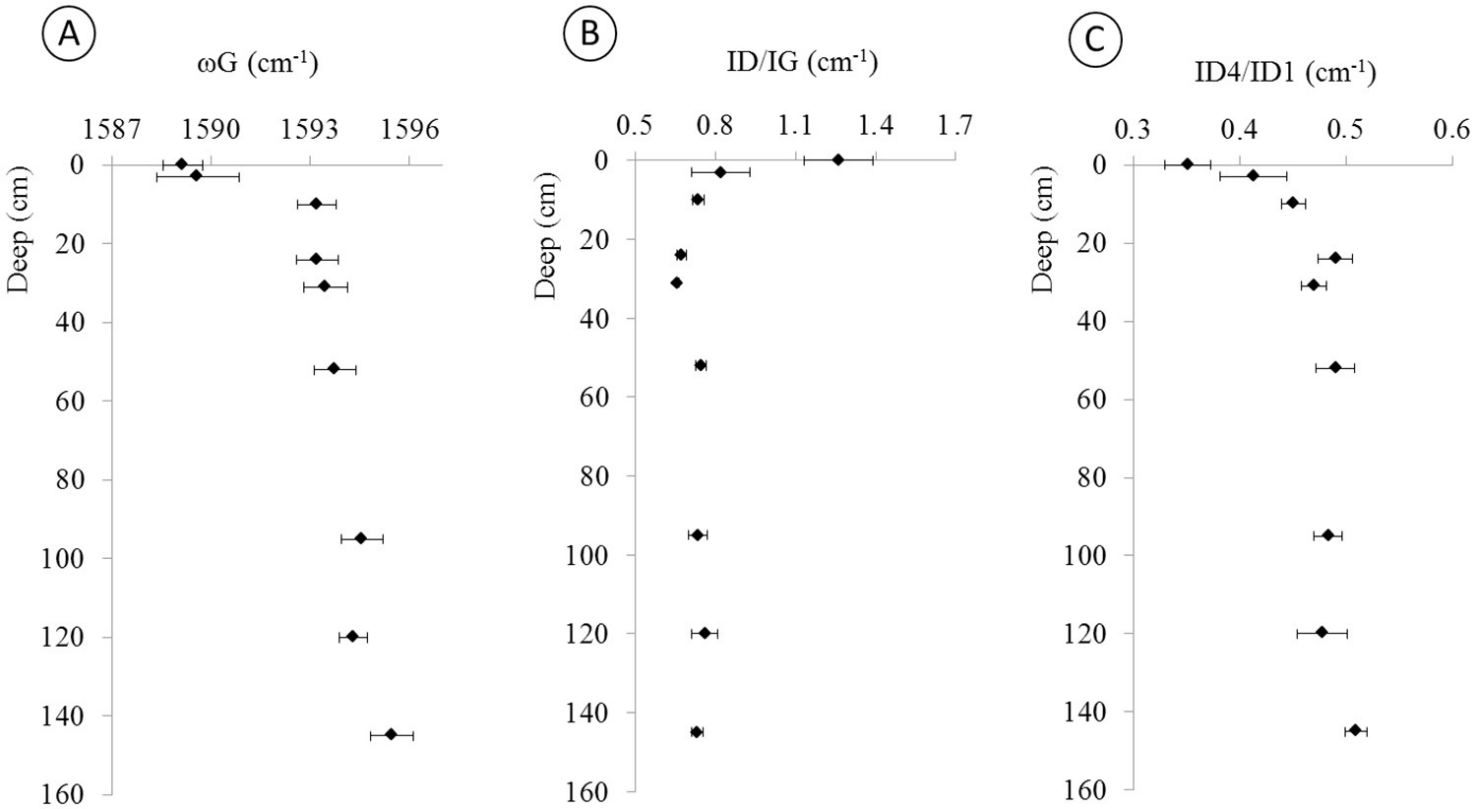
Characteristics of the Raman spectrum distributed along the stratigraphic profile in the following sequence: (A)—frequency of G (cm^-1^); (B)—intensity ratio of ID/IG; and (C)—intensity ratio of ID4/ID1.

The main characteristic of the Raman spectra of the charcoal with depth in the soil profile can be observed in Fig 6. Fig 6A illustrates that the G-band clearly undergoes a shift to regions of higher energies, with displacement from 1589 cm^-1^ to 1596 cm^-1^.

The ratio between the intensity of the D4 and D1 bands (Fig 6C) was analyzed to identify changes in lattice defects that indicate an oxidation trajectory. The results show that the D1 band (for which vibrational modes are related to lattice defects from structures with 6 carbon atoms) decrease their intensity relative to the D4 band (which is related to sp2-sp3- structural defects, CC or C = C). However, Fig 6B illustrates the decreasing ratio between the intensities of ID/IG with depth in the soil.

**Fig 7:**
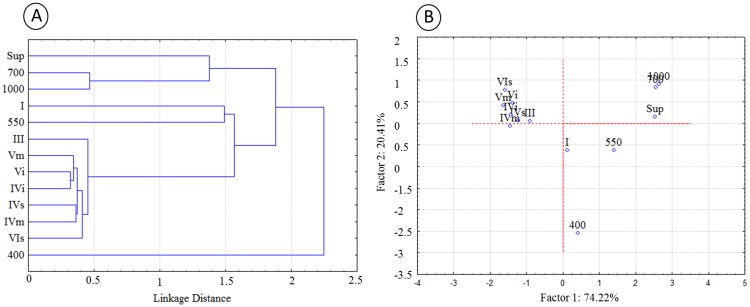
Analysis of the grouping of the samples produced in the muffle oven and collected from the archaeological site: 400, 500 and 700 °C; Sup—surface; I—layer I—610 years BP; III—layer III, 1170 years BP; IVs–layer IV-upper; IVm–layer IV-middle; IV- layer IV-lower; Vm–layer V- middle (8500 years BP); VI–V lower; VIs–layer VI upper.

The CA and PCA were performed using the variables ωG, ωD1, and the ratio of the area of the D1 band and G band (AD1/AG) was used to identify a pattern and similarities among the samples studied (Fig 7A and 7B). The sample with lower structural organization, which was pyrolyzed at 400 °C, was not closely similar to any other sample. However, the results revealed a similarity between the charcoal samples collected at the Surface layer of the archaeological site, and samples produced at higher temperatures from the pyrolysis at 700 °C and 1000 °C, which include the charcoal that was produced in muffle ovens and showed better structural organization. The similarity was greater between samples pyrolyzed at 550 °C and samples with dates older than 1670 ± 50 years BP (Layers IV, V and VI). Surface samples are relatively new (from the early 20th century), and the samples obtained from Layer I (older than the Surface) exhibit less alteration. The samples collected in Surface and Layer I (Fig 5A) have greater similarity to charcoals produced at more elevated pyrolysis temperatures (700 °C and 1000 °C).

Fig 7B indicates that two factors account for 94% of the variability of the data. Factor 1, which explains 74.22% of the variability of the data, is attributed to the charcoal organization (graphitic component) acquired during the pyrolysis process. The Surface samples are from the archaeological site Bibocas II and resemble the samples pyrolyzed at 700 and 1000 °C. The samples from Layer I resemble the samples pyrolyzed at lower temperatures (550 and 400 °C), indicating the change of carbon structure due to the oxidation processes. Factor 2, which explains 20.41% of the variability of the data, represents the unorganized fraction of the charcoal particles. In the archaeological samples, this result suggests the nanostructural evolution during the alteration processes because in fresh charcoal, this result is related to lattice defects, generated during the pyrolysis process.

### Cross-section analyses of biochar particles found in the ancient anthropic soil

**Fig 8:**
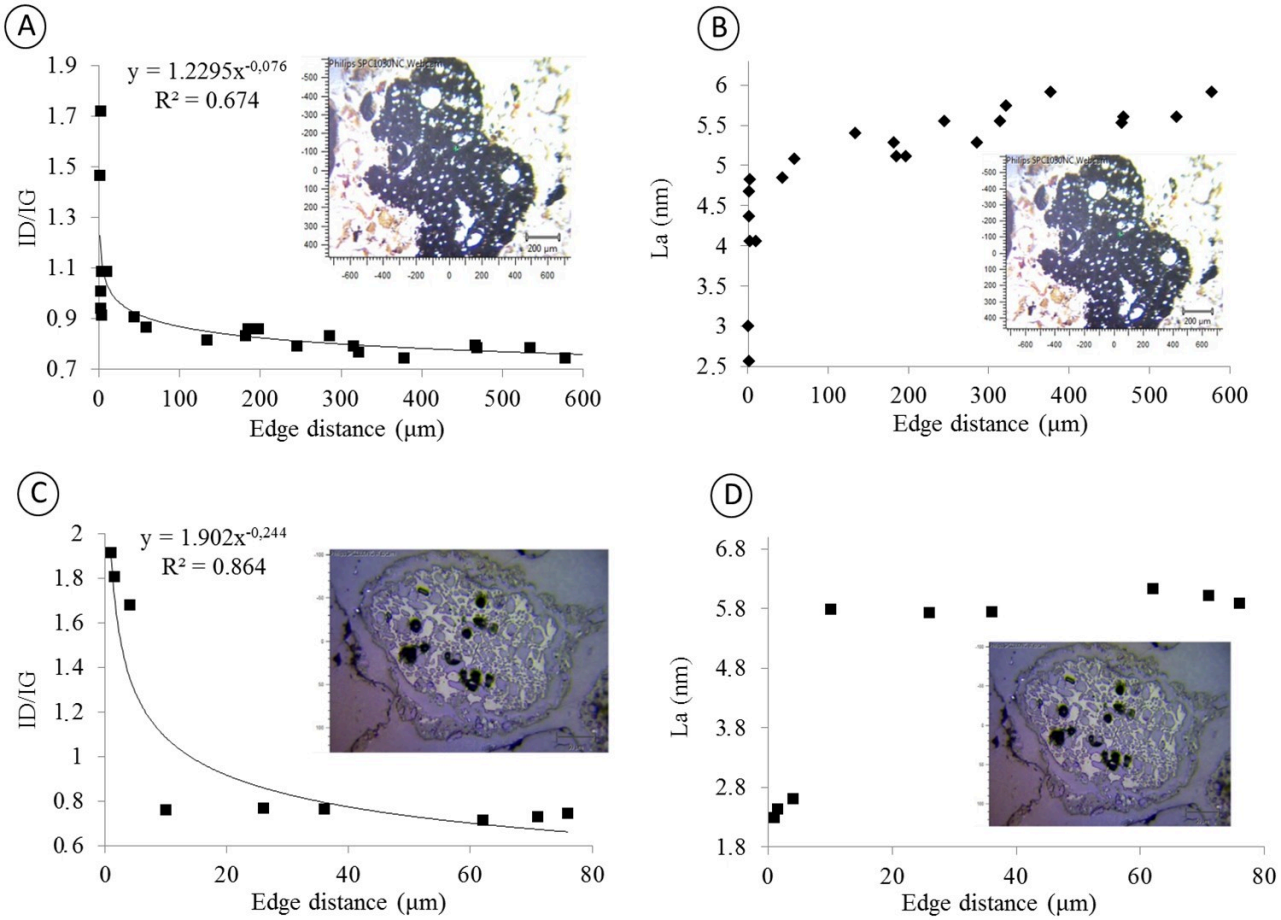
Structural disorder and size of the crystal from the periphery towards the center; A and B, ID/IG and La of charcoal at 1.5 mm; C and D, ID/IG and La of charcoal at 600 μm.

To observe how the alteration process occurs in biochar, a cross-section of two biochar particles found in the micromorphological samples was generated (soil thin sections of Layer III, Fig 1). These two particles, which had diameters of approximately 1.5 mm and 600 μm (Fig 8), were measured at distinct points starting at approximately 1.5 μm from the edge toward the center of the particle.

Fig 8A shows that smaller ratios between the intensity of bands D and G are found farther from the edge of the particle. At the first measurement point at approximately 1.5 μm away from the edge, the ID/IG ratio shows a value of 1.72, while at 44 μm from the edge, this value drops to 0.97, and at approximately 600 μm from the first measurement point, it reaches a minimum of 0.774. Higher ID/IG ratios indicate defects in the crystal lattice that affect the La, which will increase with distance from the particle edge (Fig 8B). The first measurement point at approximately 1.5 μm from the edge has a La of 2.5 nm; at 44 μm from the edge, the La calculated by the Tuinstra and Koening equation [34] was 4.85 nm, and at 600 μm from the edge, the La was 5.98 nm.

Cross-sectional analyses of the smaller particle size (600 μm) reveal a similar behavior with previous samples. The mathematical model that describes the data behavior is an exponential model. Greater structural disorder (higher ID/IG) is observed closer to the edge. The ID/IG values are 1.8 to 2 μm at the edge and decrease to 0.76 at 44 μm from the particle edge. The La values have the same behavior and are smaller at the particle edge, with a value of 2.46 nm at 2 μm from the edge and 5.74 nm at 44 μm. For the smallest sample (600 μm), the ID/IG is higher than that obtained at the same distance for the larger sample (1.5 mm). The same behavior is observed for La, with smaller charcoal particles showing greater crystal lattice disorder and a smaller La.

### SEM-EDS approach to observing the interaction between biochar particles and soil matrix

**Fig 9:**
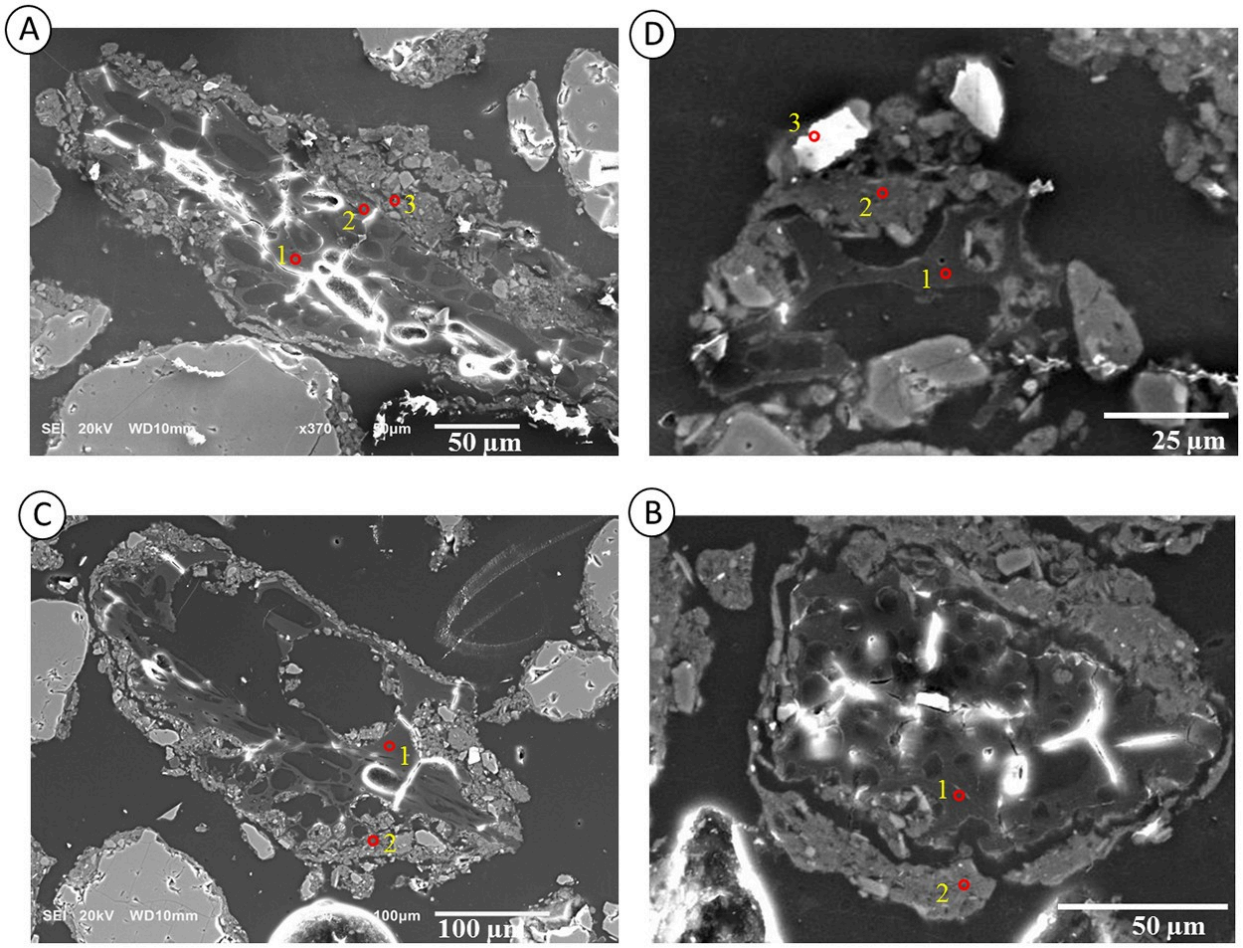
Scanning electron microscopy of particles of charcoal from a soil thin section collected between Layers II and III of the Bibocas II archaeological site. The red points indicate the EDS microanalysis data shown in Table 3. Images A and B show the charcoal particles within a soil microaggregate. Image C shows a clay coating over the charcoal particles. Image D illustrates the quasi-coating formed by clay minerals and nano-charcoal particles, which is suggested by the high content of carbon in the clay matrix.

Micromorphological samples were analyzed using a scanning electron microscope coupled to an energy-dispersive spectrometer to study the interaction of charcoal fragments with the soil matrix. Charcoal fragments without a clay coating or quasi-coating were rarely observed, and in most of the observations, the charcoal particles included a fine fraction on the periphery. The results of the SEM-EDS analyses are simplified in Fig 9 and Table 3. Fig 9A and 9C show an elongated charcoal fragment with a clay coating and a fine sand fraction on the periphery. Fig 8B shows a small microaggregate composed of charcoal in the center and the clay, silt and fine sand fractions. Fig 9D shows the formation of a microaggregate that has a charcoal fragment in its interior and presents a clay coating and quasi-coating throughout its periphery.

**Table 3:** Microanalysis points observed by EDS and indicated in Fig 9A, 9B, 9C and 9D.

Image	Point	Element (% mass)						
		C	O	Al	Fe	Si	K	P
9A	1	66.69	28.96	4.24	-	-	-	-
	2	52.15	26.58	7.28	5.24	7.75	1	-
	3	50.23	27.24	7.8	4.21	9.05	4.21	-
9B	1	62.29	32.67	3.62	0.51	0.48	0.15	0.28
	2	38.28	34.45	8.8	4.41	12.13	1.77	0.16
	3	14.88	48.58	2.12	-	33.47	0.95	-
9C	1	66.49	25.46	3.67	0.72	0.37	-	0.29
	2	3.14	37.15	8.77	5.21	10.89	0.85	-
9D	1	69.09	27.38	2.86	0.67	-	-	-
	2	62.97	21.68	5.22	3.35	6.03	0.76	-

The data in Table 3 are the results of the EDS microanalysis of the red points shown in Fig 8. The microanalysis carried out by EDS revealed that the soil clay matrix has a kaolinitic-oxidic composition because the Al:Si ratio is close to 1 for certain points (point 2 in Image 9A; point 2 in Image 9C) (Fig 9). The microanalyses illustrated in Fig 9B (point 1) show a particle and charcoal with Fe, Al, Si, and traces of K and P (Table 3), and point 2 reveals traces of K and P in the clay matrix.

Points 1 and 2 in Fig 9A are from the middle of the particle and near the edge of the charcoal, while point 3 shows the clay coating. A comparison of the data at points 1 and 2 shows that at point 1, the C:O ratio is 2.30 while at point 2, it is 1.96, which indicates a higher presence of O atoms in relation to C atoms, suggesting that there are substantial amounts of carboxylic groups at the edge of the particle. In addition, at point 2, Al, Fe, Si and traces of K are observed. Si and K are possibly inherited from the plant material itself, while Al and Fe reflect the interaction between the charcoal particle and the soil through adsorption processes.

The EDS data from Fig 9A and 9D suggest that the fine fraction, clay coating (Fig 9A) and quasi-coating (Fig 9D) are composed of a significant amount of nano-charcoals, which leads to the large amount of carbon identified in the micro-chemical analysis (points 2 and 3 of Fig 9A and 9D, respectively) that is similar to the content found in the carbon particle analysis (point 1 of Fig 9A and 9D).

## Discussion

### Structural differences between modern and fossilized charcoals and evolution of biochar structures over time

The pyrolysis process promotes the loss of hydrogen and oxygen and the formation of long molecules of carbons, and as a consequence, the carbon structures evolve into graphite structures (Fig 4A) [44]. Hemicellulose is decomposed at temperatures of approximately 202–315 °C, while cellulose decomposes between 280 and 400 °C. Lignin is a complex macromolecule with different chemical bonds, and its degradation varies according to the material that was burned and disappears at temperatures over 650 °C [57, 58]. The cellulose decomposition can be observed by the appearance of the G and D bands in the samples pyrolyzed at temperatures above 300 °C (Fig 4A), which indicates the formation of ordered ring structures. As the pyrolysis temperature increases, the structures become more ordered; this process can be shown by the shift of the G band from 1584 to 1590 cm^-1^ (Fig 4B) and the decreased FWHM of the G band (Fig 4C).

According to the elapsed time, the structural organization of the charcoal during the pyrolysis process changes due to the oxidation process, with the frequency of the G band shifting to higher energy from 1589 cm^-1^ to 1596 cm^-1^ (Fig 6) with greater depth into the soil profile. The shift of the G band to higher energy associated with a decrease of the defects of the D1 band (sp2 in rings) in relation to the D4 band (ionic impurities) suggests a charge transfer signature between sp2 structures and the soil matrix. This interaction between the biochar particles and the soil mineral matrix was previously demonstrated by Shepherd et al. [59], Archanjo et al. [31], and Hagemann et al. [60] and others [30, 61, 62].

The biochar and soil matrix interaction promotes the carbon oxidation pathway [19], which can be observed by the decreasing ID/IG ratio and the shift of ωG to a higher frequency as a function of depth in the soil profile (Fig 6A and 6B). These data show that the alteration process acts in the degradation of disorganized structures, contributing to the relative increase of the organized fraction (nanographite structures) [54, 55]. As more unstable and disordered structures are oxidized, the nanographite structures remain unaltered due to their stability. This alteration in the biochar structure occurred in approximately the first 2000 years after the initial burning (Layer III; 1698 to 1656 yr cal BP). Between 1698 and 8543 yr cal BP, the biochar particles continued to undergo changes in structure, although to a lesser extent. After the middle Layer V (8543 yr cal BP), there is an apparent stabilization of the values of the shift in ωG and ID/IG, which indicates a decreased oxidizing process because of the predominance of the nanographite component.

During the first 2000 years following the "creation" of charcoal, the most significant structural changes occurred. The amorphous phase of charcoal is the one that suffered major changes, which is indicated by the appearance of new defects in the crystalline lattice. The graphitic component underwent only minor changes and remained more stable, although there was a decrease of the La. Finally, the next 9000 years did not bring about significant changes in the nanographite structure, although there was a considerable blue-shift in the G band, which suggests that there is an amorphization trajectory or an interaction between the soil matrix that caused charge changes. Previous works on a much smaller timescale also detected changes in the amorphous component of biochar particles, although the researchers did not detect any alterations in the graphite component [20, 63].

### Structural alteration preferentially occurs on the charcoal periphery

According to the data shown in Figs 8 and 9, particles with a diameter of 1.5 mm and 600 μm have a high structural disorder on their periphery that decreases toward the center of the particle. This characteristic was also found for Indian Black Earth of the Amazon region [12, 33]. The data in Fig 8 also indicate that smaller particles exhibit greater structural disorder, both on the periphery and at the center. This difference in structural order/disorder according to the size of the charcoal particle indicates the existence of physical protection against oxidation processes. Thus, the protection process against structural alteration is more efficient for larger particles [64].

From the moment that the plant material is pyrolyzed, the carbon presents molecular and structural characteristics, with the sp2/sp3 ratio determined by the characteristics of the material pyrolyzed (e.g., lignin and cellulose), temperature and pyrolysis [48, 65]. It is worth mentioning that in archaeological sites, it is common for bonfires to be placed in locations that previously hosted bonfires. Therefore, charcoal from archaeological sites can undergo several processes of pyrolysis until they are buried by sedimentary processes.

Once the sedimentary action has buried the charcoal, the main process the particles undergo is biological oxidation [64], which will "consume" the more unstable fraction of carbon and oxidize aromatic rings into carboxylic and phenolic groups. Thus, the Surface layer will have a higher capacity for adsorbing chemical elements because of the oxidation-generated charges as well as the high specific surface associated with the small La of approximately 2.3 nm, which was estimated by the Tuinstra and Koenig equation [34] (Fig 9B and 9D). This high specific surface area and small La will promote the adsorption of Fe, Al, K, Ca, and P onto biochar particles as demonstrated by Archanjo [31] and Shepherd [59]. When the soil solution flows between the pores of the soil, the charcoal particles will encounter a basic microenvironment that favors the precipitation of certain molecules, such as Fe oxides and carbonates. Fe oxides may contribute to the adsorption of phosphorus [66], potassium, and other nutrients [67] and pollutants [68, 69]. Hagemann [60] proposed that not only the interaction of the mineral matrix and biochar particles is responsible for nutrient adsorption but also highlighted the action of organic clusters with biochar and organic compounds.

The interaction of the charcoal with the soil is clearly observed in Fig 8. In Fig 8A and 8C, an elongated charcoal fragment with a clay coating is observed. Fig 8B shows a microaggregate composed of charcoal in the center with a clay coating and the presence of quartz in the silt fraction. The interaction between the soil matrix and charcoal particles can form soil microaggregates (Fig 8D) with a charcoal fragment at the core and clay forming a coating and quasi-coating throughout the periphery.

The oxidation of the charcoal is due to microbiological action as well as the reduction and oxidation processes that occur with the soil constituents, such as with the Fe oxides on the surface of the charcoal [14, 59]. These redoximorphic reactions consume electrons coming from the periphery of the charcoal particle, thus aiding its weathering and contributing to its physical fragmentation. These interactions occur with the ion-exchange and electrons in soil and charcoal and explain the G-band displacement shown in Fig 6A. However, this "encrustation" of the charcoal particle by oxides and clayey material as observed in Fig 8A–8D as well as in Shepherd [59] and Sousa [40] will contribute to its permanence in the environment due to the physical protection provided by the mineral fraction [5]. Instead of the carbon being oxidized, the Fe oxides incorporated in the surface of the particle will undergo oxidation-reduction processes, thus promoting the physical and chemical protection of the charcoal particle. In addition to iron oxide, other constituents may exert such physical protection, such as Al, Mn, Si and other clay constituents.

## Conclusions

This work supports previous findings that show that charcoal will suffer alteration processes over time, and our results support the following conclusions. i) The most significant changes occur in the crystalline lattice during the first 2000 years following the contact of the charcoal particle with the soil matrix. ii) The amorphous fraction is more altered, which is reflected in the decreased number of defects associated with the D1 band in relation to the D4 band. The D1 band defects are related to sp2 bonds on amorphous carbons, and the D4 band defects are associated with ionic impurities, which indicate the interaction between the biochar particle and soil matrix. iii) Changes in the graphite structure occur after the initial 2000 years, with a decreased oxidizing process and a predominance of the nanographite component. iv) Smaller biochar particles have a smaller La and a greater edge density and reactivity.

The application of Raman spectroscopy and a long timescale perspective have provided novel information on the chemical structure of charcoal that is difficult to obtain by other means. The results provide insights on the evolution of biochar structures and the dynamic alteration of biochar over time from the end of the Pleistocene (12702 to 12543 yr cal BP) to the historical period (beginning approximately 500 years ago).

The data presented here confirm the hypothesis that the presence of biochar in the environment is due to its graphite structure and allow us to conclude that graphite structures can change over 12000 years and the interaction between biochar particles and soil matrix produces defects (D4 band) in the crystalline lattice.

## Supporting information

S1 Fig Plot of linkage distances across stepd (euclidian distances) of cluster analysis. (TIF)

S1 Table Factor scores, based on correlations, of principal component analysis. (DOCX)

S2 Table Case contributions, based on correlations, of principal component analysis. (DOCX)
