# Supplementary figures and images for: Dynamic of the structural alteration of biochar in ancient Anthrosol over a long timescale by Raman spectroscopy
Source: PLoS One. 2020 Mar 23;15(3):e0229447. doi: 10.1371/journal.pone.0229447 (PMC7089566; doi:10.1371/journal.pone.0229447)

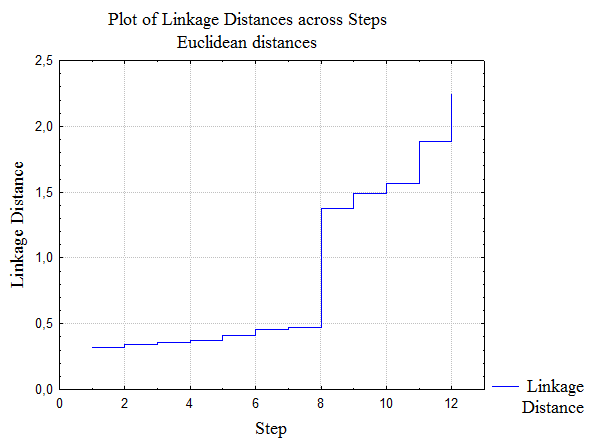

Supplement: S1 Fig — (TIF) [file pone.0229447.s001.tif]
