# Supplementary material for: Dynamic of the structural alteration of biochar in ancient Anthrosol over a long timescale by Raman spectroscopy
Source: PLoS One. 2020 Mar 23;15(3):e0229447. doi: 10.1371/journal.pone.0229447 (PMC7089566; doi:10.1371/journal.pone.0229447)
Supplement: S1 Table — (DOCX) [file pone.0229447.s002.docx]

**S1 Table.** **Factor scores, based on correlations, of Principal Component Analysis**

| Case | Factor 1 | Factor 2 |
| --- | --- | --- |
| Sup | 1,468126 | 0,16505 |
| I | 0,073122 | -0,69204 |
| III | -0,525648 | 0,05720 |
| IV s | -0,713954 | 0,07559 |
| IV m | -0,837673 | -0,07280 |
| IV i | -0,834185 | 0,22081 |
| Vs | -0,924501 | 0,85967 |
| Vm | -0,955263 | 0,45259 |
| Vi | -0,805381 | 0,51954 |
| 400 | 0,235745 | -2,80965 |
| 550 | 0,811501 | -0,69352 |
| 700 | 1,478321 | 0,91750 |
| 1000 | 1,529789 | 1,00004 |
