# Supplementary material for: Dynamic of the structural alteration of biochar in ancient Anthrosol over a long timescale by Raman spectroscopy
Source: PLoS One. 2020 Mar 23;15(3):e0229447. doi: 10.1371/journal.pone.0229447 (PMC7089566; doi:10.1371/journal.pone.0229447)
Supplement: S2 Table — (DOCX) [file pone.0229447.s003.docx]

**S2 Table. Case contributions, based on correlations, of Principal component Analysis**

| Case | Factor 1 | Factor 2 |
| --- | --- | --- |
| Sup | 17,96161 | 0,22702 |
| I | 0,04456 | 3,99105 |
| III | 2,30255 | 0,02726 |
| Ivs | 4,24775 | 0,04762 |
| Ivm | 5,84747 | 0,04416 |
| Ivi | 5,79887 | 0,40631 |
| Vs | 7,12252 | 6,15864 |
| Vm | 7,60439 | 1,70698 |
| Vi | 5,40532 | 2,24936 |
| 400 | 0,46313 | 65,78436 |
| 550 | 5,48778 | 4,00803 |
| 700 | 18,21195 | 7,01513 |
| 1000 | 19,50211 | 8,33408 |
